# Supplementary material for: Insulin deficiency inhibits vaccine-mediated antibody and germinal center B-cell formation in mice
Source: bioRxiv. 2025 Sep 26:2025.09.24.677144. Preprint. [Version 1] doi: 10.1101/2025.09.24.677144 (PMC12485872; doi:10.1101/2025.09.24.677144)
Supplement: Supplement 3 [file media-3.pdf]

ONLINE-ONLY SUPPLEMENTAL MATERIAL

FOR

Insulin deficiency inhibits vaccine-mediated antibody and germinal center B-cell formation in mice.

Christopher J. Genito, Pablo Ariel, Mark T. Heise, Lance R. Thurlow

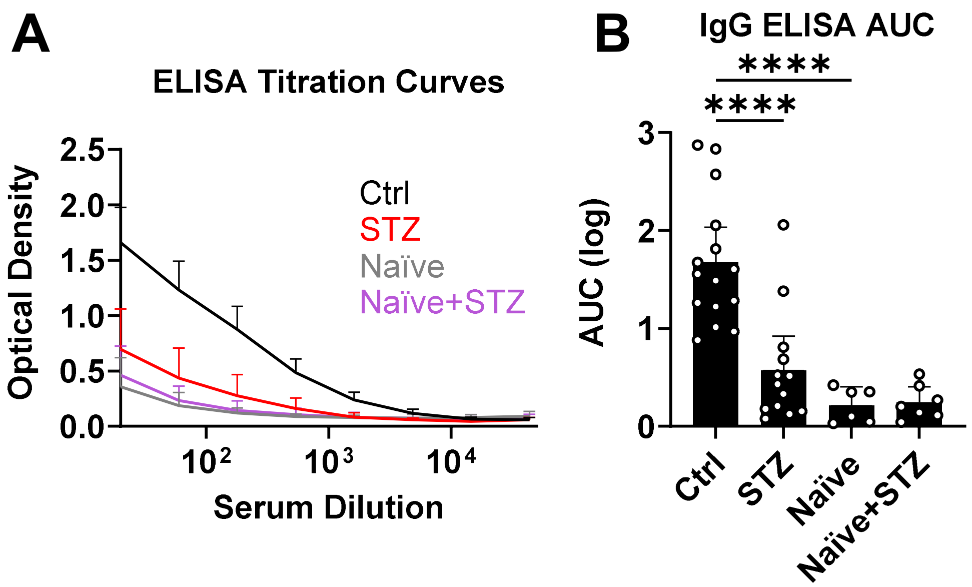

Figure S1. Additional antibody measurements by ELISA. Mice were made insulin-deficient using streptozotocin (STZ) and immunized with ovalbumin (Ova) adjuvanted with alum. Two weeks after immunization, Ova-specific IgG antibody levels were quantified by ELISA for control (Ctrl; n = 15), STZ-treated (n = 13), and naïve control (naïve, n = 6), and naïve STZ-treated (naïve+STZ, n = 7) mice. **(A)** Each group's mean (+95% clearance interval) optical density values (optical density at 450 nm wavelength corrected with 570 nm background subtraction) obtained from ELISA at each tested serum dilution. **(B)** Area under the curve (AUC) calculated for each animal's individual ELISA titration curve, using log-transformed serum dilutions. Data are presented as mean + 95% clearance interval. ****$p < 0.0001$, ANOVA with Tukey's test for multiple comparisons. Data is pooled from three independent experiments.

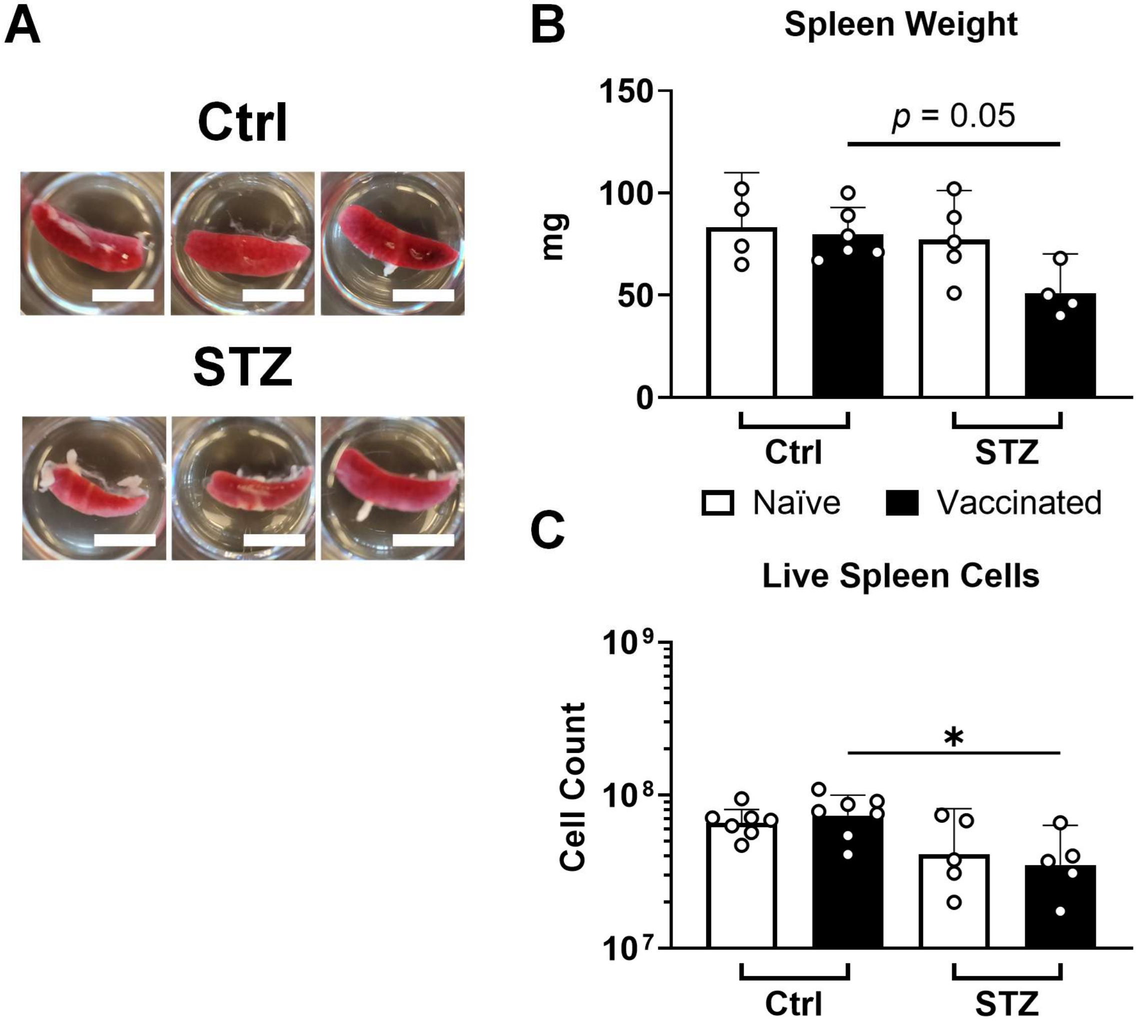

Figure S2. Spleen size from vaccinated insulin-deficient mice. Spleens were removed 2 weeks after vaccination with ovalbumin adjuvanted with alum from mice made insulin-deficient through streptozotocin administration (STZ) or from control animals (Ctrl). **(A)** Representative images of removed spleens. Scale bar = 15 mm. **(B)** Spleen weights (Ctrl: naïve n = 4, vaccinated n = 6; STZ: naïve n = 5, vaccinated n = 4). Data are represented as mean + 95% CI. **(C)** Cells were isolated from spleens and live cell counts were determined by staining with trypan blue (Ctrl: naïve n = 7, vaccinated n = 7; STZ: naïve n = 5, vaccinated n = 5). Data are represented as geometric mean + 95% CI. Statistical comparisons were made by ANOVA with Tukey's correction for multiple comparisons. *$p < 0.05$. Data was pooled from 2 independent experiments.

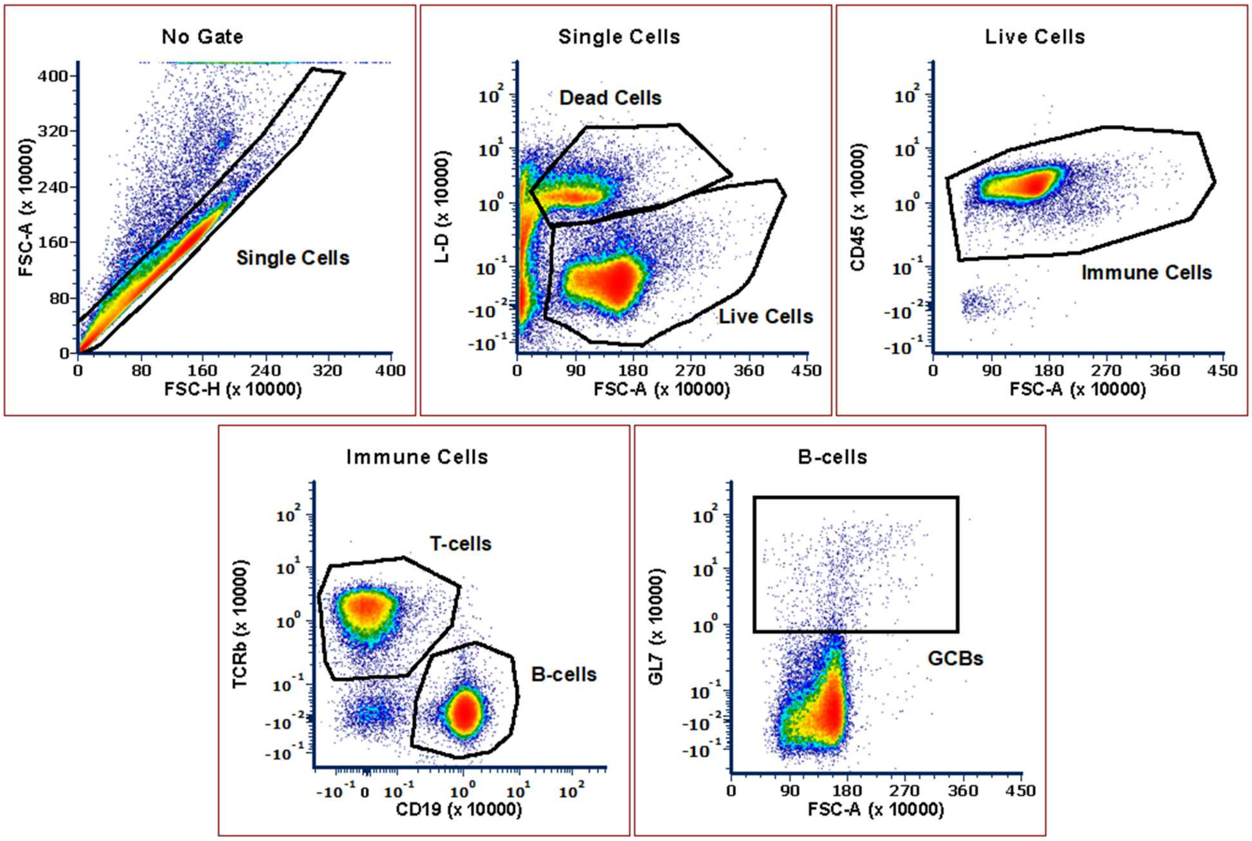

Figure S3. Gating strategy for immune cell subset analysis by spectral flow cytometry.

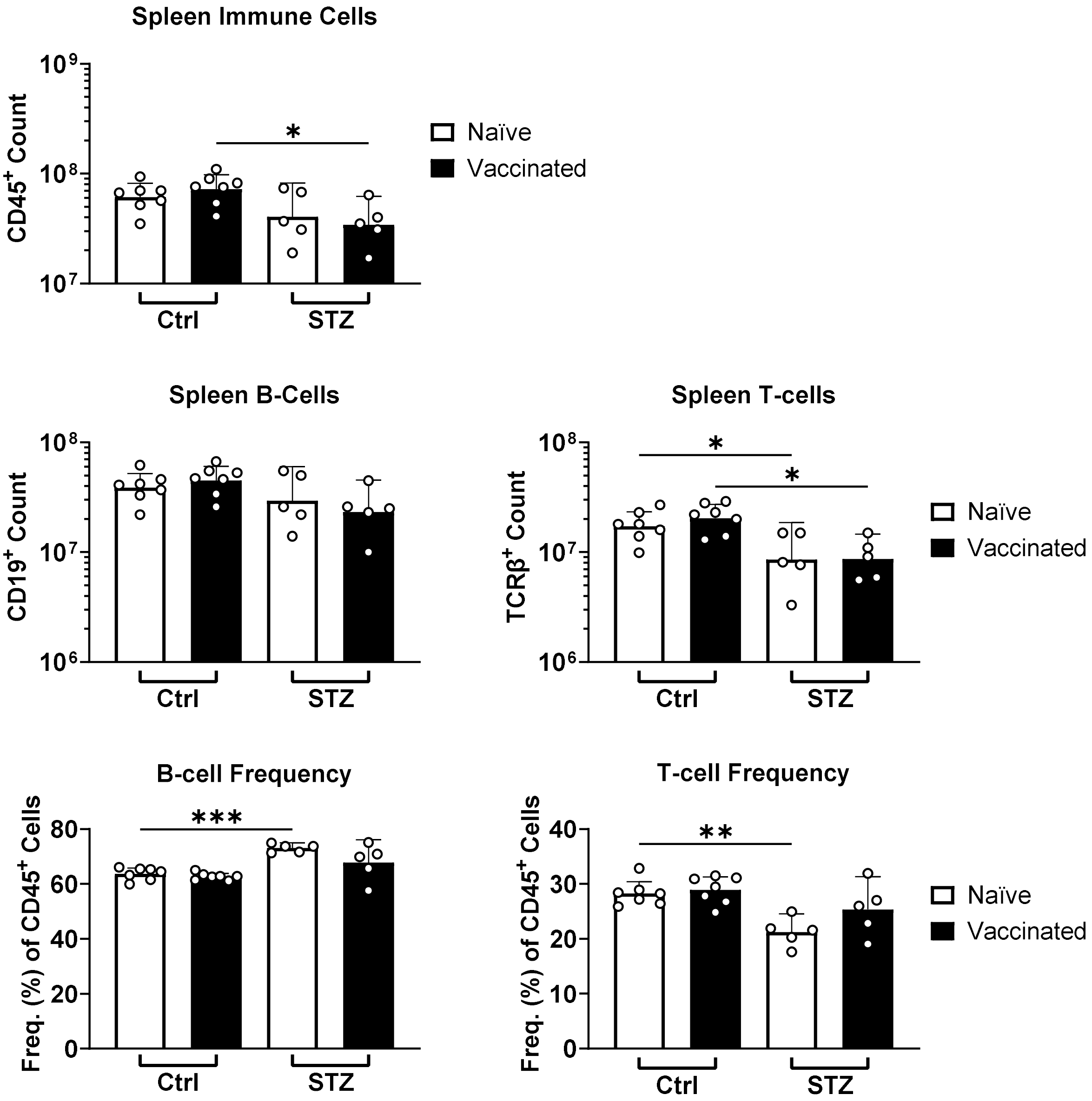

Figure S4. Immune cell counts and frequencies in spleens after vaccination in insulin-deficient mice. Total immune cell **(A)**, total B-cell **(B)**, and total T-cell **(C)** counts, as well as B-cell **(D)** and T-cell **(E)** frequencies among total immune cells, were quantified by flow cytometry in spleens 2 weeks after vaccination with ovalbumin adjuvanted with alum for insulin-deficient (STZ, naïve n = 5, vaccinated n = 5) and control (Ctrl, naïve n = 7, vaccinated n = 7) mice. Data is represented as geometric mean + 95% CI. Statistical comparisons were made using ANOVA with Tukey's correction for multiple comparisons. *$p < 0.05$, **$p < 0.01$, ***$p < 0.001$. Data was pooled from 2 independent experiments.

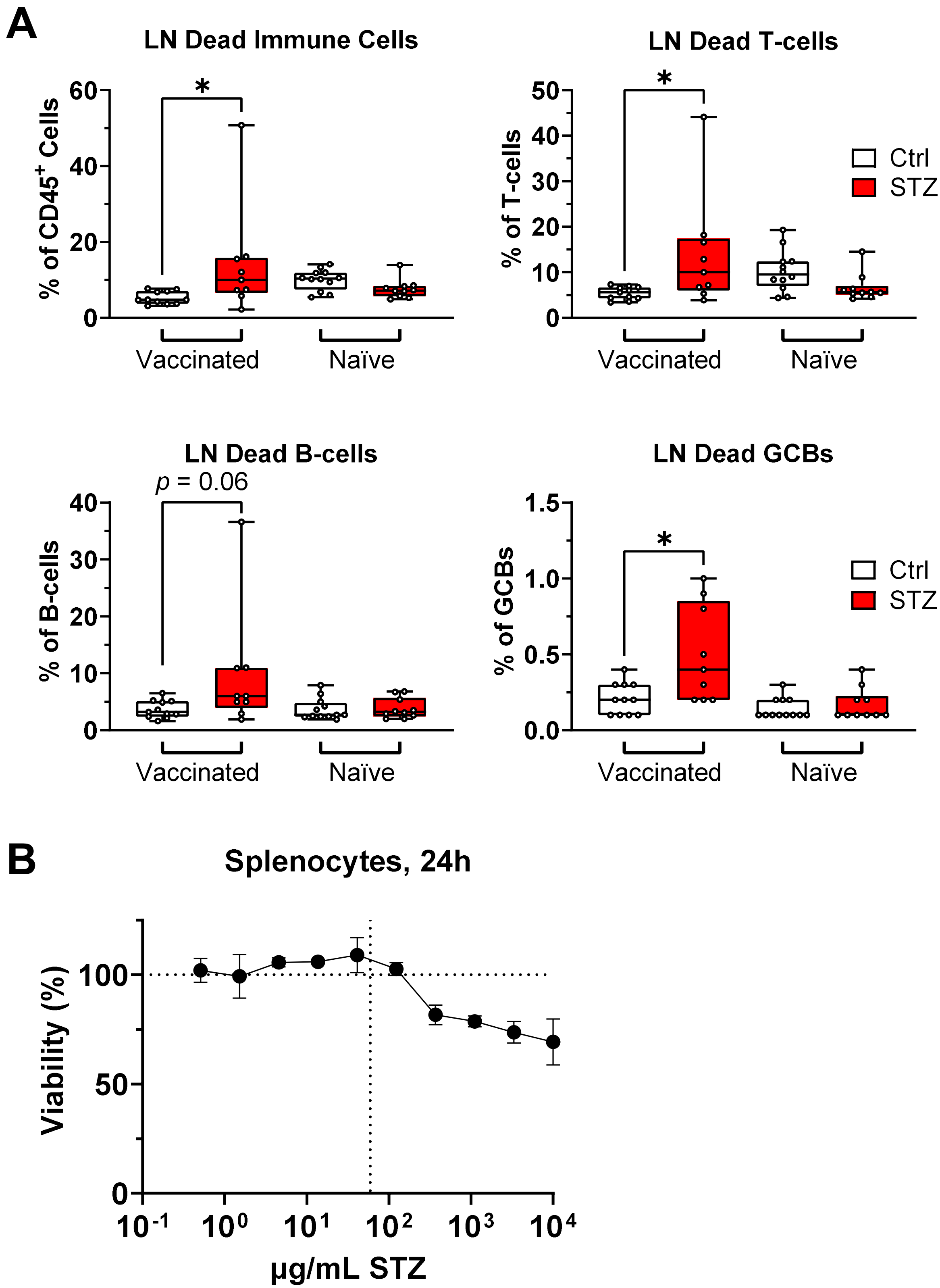
A
LN Dead Immune Cells
% of CD45+ Cells
*
Vaccinated
Naïve
LN Dead T-cells
% of T-cells
Ctrl
STZ
LN Dead B-cells
p = 0.06
% of B-cells
LN Dead GCBs
% of GCBs
B
Splenocytes, 24h
Viability (%)
µg/mL STZ

Figure S5. Streptozotocin treatment-related immune cell death. (A) Frequency (%) of dead cells in each immune cell population analyzed in draining lymph nodes (LN) 2 weeks after vaccination (ovalbumin and alum) by flow cytometry. Data are presented as quartiles with the center line at the median (control mice: naïve n = 12, vaccinated n = 11; STZ-treated mice: naïve n = 10, vaccinated n = 9). Vaccinated groups were statistically compared by Mann-Whitney *U* test. $^{*}p < 0.05$. GCBs = germinal center B-cells. Data was pooled from 3 independent experiments. **(B)** Immune cells were isolated from the spleen of a control mouse and cultured ex vivo in the presence of streptozotocin (STZ) at various concentrations for 24h. Viability was determined by extracellular LDH detection, compared to untreated cells. Vertical dotted line represents the highest plasma concentration of STZ detected in mice 3 h after STZ administration reported by Anderson et al. (29). Each point represents mean and SD (n=3).

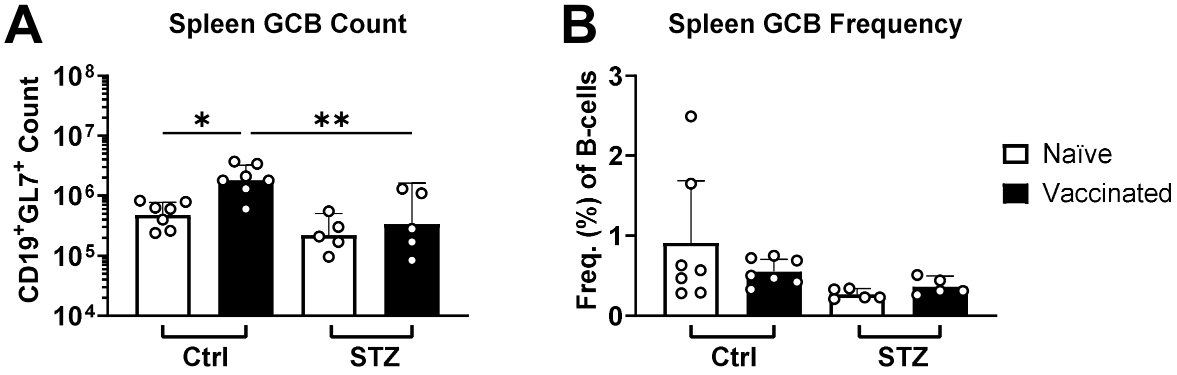

Figure S6. Splenic germinal center B-cells (GCB) after vaccination in insulin-deficient mice. GCB counts **(A)** and GCB frequencies among total B-cells, were quantified by flow cytometry in spleens 2 weeks after vaccination with ovalbumin adjuvanted with alum for insulin-deficient (STZ, naïve n = 5, vaccinated n = 5) and control (Ctrl, naïve n = 7, vaccinated n = 7) mice. Data is represented as geometric mean + 95% CI. Statistical comparisons were made using ANOVA with Tukey's correction for multiple comparisons. *$p < 0.05$, **$p < 0.01$. Data was pooled from 3 independent experiments.

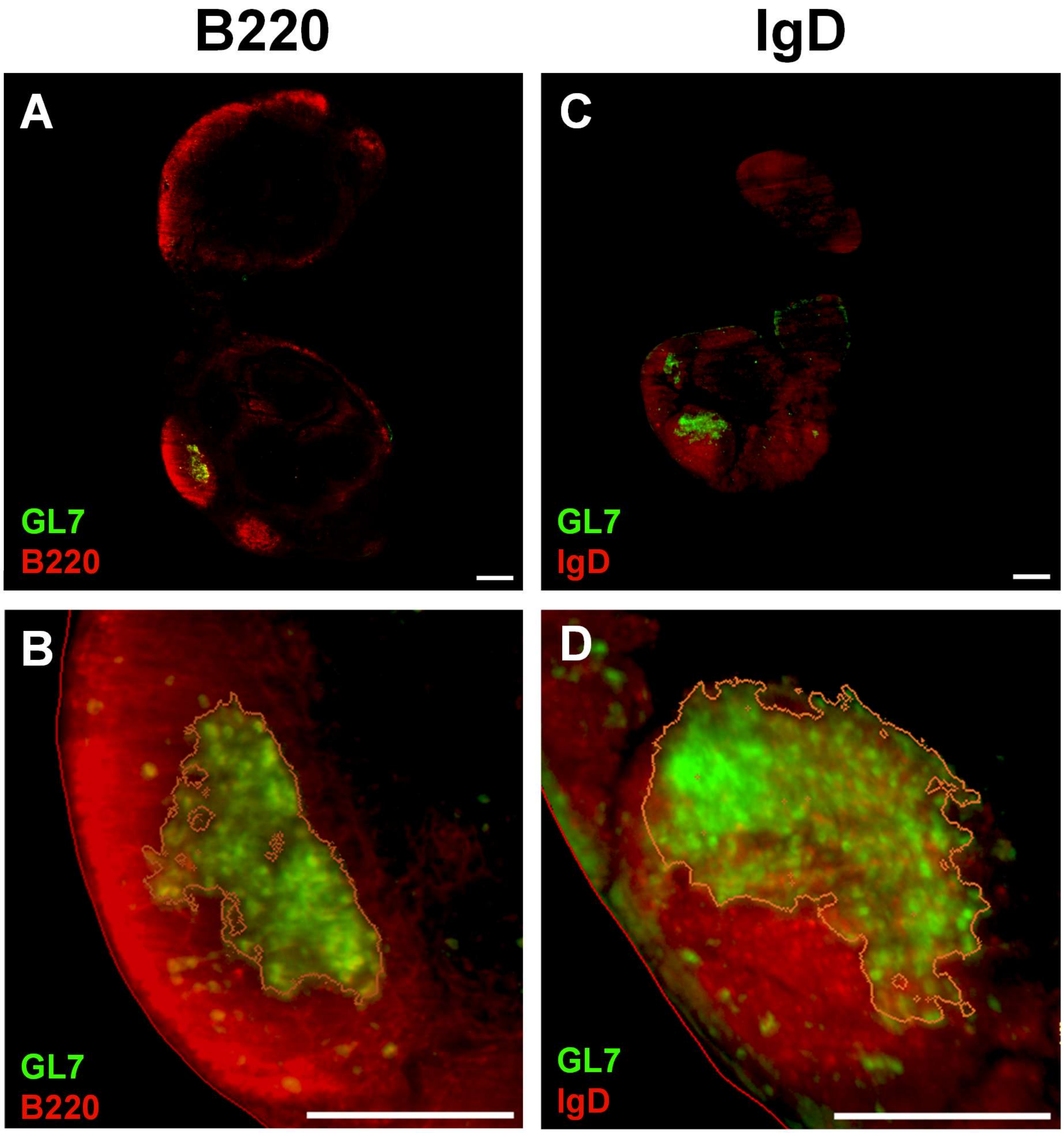

Figure S7. B-cell follicle marker comparison. 2D slice images from light sheet microscopy of draining inguinal lymph nodes 2 weeks after vaccination with ovalbumin adjuvanted with alum. Germinal center B-cells were detected by GL7 (green) and B-cell follicles (red) were detected either by B220 **(A,B)** or IgD **(C,D)**. In B and D, the red line represents the outside edge of the lymph node, and the orange line represents the edge of the germinal center as determined by analysis in syGlass. Scale bar 200 μm.

Table S1. Antibodies and related conjugations used for spectral flow cytometry and light sheet microscopy.

Target	Conjugate	Vendor	Clone/ID	Use	Dilution
CD45	PerCP/Cy5.5	BioLegend	30-F11	Flow	1:1600
GL7 (Ly77)	PE	BioLegend	GL7	Flow	1:800
CD19	BV570	BioLegend	6D5	Flow	1:200
TCRβ	BUV395	BD	H57-597	Flow	1:100
Rat IgG	AF647	Jackson ImmunoResearch	212-606-168	Light sheet	1:400
B220 (CD45R)	Unlabeled	Invitrogen	RA3-6B2	Light sheet	1:800
GL7	Biotin	BioLegend	GL7	Light sheet	1:200
Biotin (via streptavidin)	AF568	Invitrogen	S11226A	Light sheet	1:1000
IgD	AF647	BioLegend	11-26c.2a	Light sheet	1:100

Table S2. Light sheet microscope settings per sample. Chromatic correction was used. A-F = empty channel used to capture autofluorescence.

Sample Name	Light Sheet Source	Number of Light Sheets	Laser Position Relative to Sample	Sheet NA	Sheet Width	Sheet Thickness (µm)	Zoom (X)	Z-spacing (µm)	Pixel Size (XY µm)	Cell Markers		
										488 nm	561 nm	647 nm
**Ctrl 1**	Right	3	Middle	0.039	100%	7.1	3.2	3.55	0.95	N/A	GL7	B220
**Ctrl 2**	Right	3	Middle	0.032	100%	7.1	2	5	1.54	N/A	GL7	B220
**Ctrl 3**	Right	3	Middle	0.024	100%	11.5	2.5	5	1.22	A-F	GL7	B220
**Ctrl 4**	Left	3	Middle	0.024	100%	11.5	2.5	5	1.22	A-F	GL7	IgD
**STZ 1**	Right	3	Slight right	0.032	100%	8.7	3.2	4.35	0.94	A-F	GL7	B220
**STZ 2**	Right	3	Middle	0.039	100%	7.1	3.2	3.55	0.94	A-F	GL7	B220
**STZ 3**	Right	3	Middle	0.032	100%	8.7	3.2	4.35	0.94	A-F	GL7	B220
**STZ 4**	Left	3	Slight left	0.032	100%	8.7	3.2	4.35	0.94	A-F	GL7	B220

Table S3. VR settings for GC identification.

Sample	Voxel Size (X-Y-Z µm)	Performance	Brightness	Contrast	Window min	Window max	Threshold min	Threshold max
**Ctrl 1**	0.95. x 0.95 x 3.55	10.00	388.5	2.741	0	17863	1452	65535
**Ctrl 2**	1.54 x 1.54 x 5.00	10.00	8.256	1.035	0	40615	1254	65535
**Ctrl 3**	1.22 x 1.22 x 5.00	3.000	1.000	1.000	0	16191	827	65535
**Ctrl 4**	1.22 x 1.22 x 5.00	3.000	1.464	0.813	296	44204	699	65535
**STZ 1**	0.94 x 0.94 x 4.35	10.00	0.287	1.000	257	40394	2485	65535
**STZ 2**	0.94 x 0.94 x 3.55	7.753	38.62	1.438	2	28527	1601	65535
**STZ 3**	0.94 x 0.94 x 4.35	8.719	5.185	1.128	587	65535	1839	65535
**STZ 4**	0.94 x 0.94 x 4.35	3.000	12.55	1.231	0	65535	1741	65535
